# The TikTok Addiction Scale: Development and validation

**DOI:** 10.3934/publichealth.2024061

**Published:** 2024-12-11

**Authors:** Petros Galanis, Aglaia Katsiroumpa, Ioannis Moisoglou, Olympia Konstantakopoulou

**Affiliations:** 1 Clinical Epidemiology Laboratory, Faculty of Nursing, National and Kapodistrian University of Athens, Athens, Greece; 2 Faculty of Nursing, University of Thessaly, Larissa, Greece

**Keywords:** TikTok, addiction, scale, tool, instrument, validation, development

## Abstract

**Background:**

There is an absence of valid and specific psychometric tools to assess TikTok addiction. Considering that the use of TikTok is increasing rapidly and the fact that TikTok addiction may be a different form of social media addiction, there is an urge for a valid tool to measure TikTok addiction.

**Objective:**

To develop and validate a tool to measure TikTok addiction.

**Methods:**

First, we performed an extensive literature review to create a pool of items to measure TikTok addiction. Then, we employed a panel of experts from different backgrounds to examine the content validity of the initial set of items. We examined face validity by performing cognitive interviews with TikTok users and calculating the item-level face validity index. Our study population included 429 adults who have been TikTok users for at least the last 12 months. We employed exploratory and confirmatory factor analysis to examine the construct validity of the TikTok Addiction Scale (TTAS). We examined the concurrent validity by using the Bergen Social Media Addiction Scale (BSMAS), the Patient Health Questionnaire-4 (PHQ-4), and the Big Five Inventory-10 (BFI-10). We used Cronbach's alpha, McDonald's Omega, Cohen's kappa, and intraclass correlation coefficient to examine reliability.

**Results:**

We found that the TTAS is a six-factor 15-item scale with robust psychometric properties. Factor analysis revealed a six-factor structure, (1) salience, (2) mood modification, (3) tolerance, (4) withdrawal symptoms, (5) conflict, and (6) relapse, which accounted for 80.70% of the total variance. The concurrent validity of the TTAS was excellent since we found significant correlations between TTAS and BSMAS, PHQ-4, and BFI-10. Cronbach's alpha and McDonald's Omega for the TTAS were 0.911 and 0.914, respectively.

**Conclusion:**

The TTAS appears to be a short, easy-to-use, and valid scale to measure TikTok addiction. Considering the limitations of our study, we recommend the translation and validation of the TTAS in other languages and populations to further examine the validity of the scale.

## 1. Introduction

Social media platforms have gained immense popularity worldwide, with an estimated five billion people using these platforms for connection, communication, and information seeking. The number of social media users has increased worldwide from 2.7 billion in 2017 to 5.2 billion in 2023, a number projected to approach six billion in 2028. Moreover, today, the average daily social media usage is 151 minutes, while in 2012 was 90 minutes [1]. High levels of digital addiction raise concerns about appropriate internet use. In particular, a meta-analysis including data from 64 countries found that the global pooled prevalence for social media addiction is 17.4%, for internet addiction is 14.2%, for smartphone addiction is 27%, for cybersex addiction is 8.2%, and for game addiction is 6% [2]. Another meta-analysis included 63 studies from 32 nations and found that the pooled prevalence of social media addiction is 24% worldwide, 31% in collectivist nations, and 14% in individualist nations [3]. There is an ongoing debate over the negative consequences of social media usage. The excessive use of social media is becoming a significant public health issue due to its association with various problems, such as depression, stress, anxiety, psychological distress, low self-esteem, impulsivity, suicide risk, work impairments, and poor sleep quality [4]–[12].

TikTok has become one of the world's most widely used applications for short-form videos, and it has over 1.5 billion active users globally every month. Therefore, the prevalence of TikTok usage globally is about 18.5%. For instance, TikTok has over 150 million monthly active users in the USA, corresponding to a prevalence of about 44.8%. Also, the prevalence of TikTok usage in Europe is approximately 32.1%, with more than 240 million monthly active users. TikTok users have grown exponentially from 133 million in 2018 to over 1.7 billion in 2024. Today, the average daily TikTok usage is 59 minutes, while in 2019 it was 27 minutes. TikTok was downloaded more than 2 billion times in 2021, and most users are adolescents and young adults (16–35 years old) [13]. In Greece, more than 3.5 million people use TikTok, corresponding to a prevalence of about 35%. Among these users, 33% are aged 13–25 years old. Moreover, in Greece, TikTok is the favorite social media application of around 30% of children and young adults (ages 13–25 years old). Additionally, 36% of TikTok users aged 13–17 years spend two hours daily on the platform, while 18.6% engage with it for more than three hours each day [14].

Launched internationally in 2017, TikTok is a free social media platform that enables users to create, edit, and share brief video clips that are enhanced with filters and feature the latest music trends. Users can download the application to their smartphones and record videos that are less than three minutes in length. TikTok offers a variety of features, including adding audio and images, live broadcasting, and earning income based on the number of followers. Unlike other social media platforms (e.g., Facebook, Twitter, Instagram, and Snapchat), which focus primarily on images and text, TikTok emphasizes brief videos [15]. TikTok's focus on short videos and interactive content has made it a popular choice for users seeking quick, engaging entertainment. Users can earn income and attract followers by producing content, making the application even more appealing. TikTok users prefer the application for various reasons, including social acceptance, comfort, and satisfaction [16].

TikTok empowers users to capture cherished memories and produce concise videos to document their lives, providing ample entertainment. However, it is also emerging as a novel form of social media addiction [17]. Social media addiction refers to the recurrence of addiction-like symptoms or a lack of self-restraint in relation to social media [18],[19]. Although research on social media addiction has concentrated predominantly on Facebook, Instagram, and other well-established social media platforms, it has overlooked TikTok's influence and the associated maladaptive behaviors [17]. Examining the behavior of TikTok addiction is essential for several reasons. First, TikTok has rapidly grown to become one of the most popular applications, surpassing other social media platforms in terms of user numbers and usage intensity [15]. Second, TikTok boasts an advanced algorithm system, particularly in terms of participation, content, and types of interaction, which makes TikTok addiction more severe than other social media platforms [17],[20],[21]. While the underlying negative effects of addiction are similar across different platforms, the intensity and driving factors of TikTok addiction are unique [17]. Third, TikTok's target audience is adolescents and young adults with short attention spans. This has raised serious concerns as TikTok addiction is seriously affecting young people.

TikTok's impact on users differs significantly from other social media platforms. While Facebook and Instagram primarily display content from followed accounts, TikTok's micro-video format centers on an endless scroll feature, delivering algorithm-driven recommendations of entertaining videos from various unknown creators. The platform's content-detection algorithm is specifically designed and continuously improved to align with users' interests, aiming to maximize their time spent on the application. This user-generated content proves highly addictive for individuals who essentially visit TikTok to immerse themselves in strangers' lives [21]–[24]. TikTok stands apart from other social networks because users' feeds are not based on conscious selections of desired content. Instead, artificial intelligence presents content and utilizes user reactions (likes, comments, and reshares) to determine potential preferences, creating a continuous cycle that begins with initial use and becomes more precise with ongoing engagement [21]–[24]. Furthermore, TikTok's core appeal lies in its ability to provide consistent dopamine releases. In simple terms, frequent TikTok usage is directly connected to the stimulation of dopamine, a pleasure or reward system. This reward is continuously produced in the brain as a response to engaging videos. It triggers a dopamine surge in the bloodstream, a feel-good hormone, and establishes a loop that encourages users to watch more videos for the same effect. Consequently, users often spend extended periods on TikTok as their brains crave the ongoing dopamine rush [25],[26].

In this context, valid measurement of social media addiction/disorder/problematic use is crucial to identify high-risk individuals. A recent scoping review found that there are 37 instruments that measure negative social networking use [27]. The Bergen Facebook Addiction Scale (BFAS) is the most widely used instrument for measuring a negative use of social media [28]. Several adaptations from the BFAS have been developed, such as the Bergen Social Media Addiction Scale [29]. Other popular instruments include the Social Media Disorder Scale [30], the Facebook Intrusion Questionnaire [31], the Generalized Problematic Internet Use Scale [32], and the Internet Addiction Test [33]. Among these instruments, four of them have been translated and validated in Greek. In particular, Dadiotis et al. [34] translated and validated the BFAS to measure levels of social media addiction in a sample of university students, Floros and Siomos [35] translated and validated the Online Cognitions Scale to measure internet addiction in a sample of adolescents aged 12–18 years old, Xanidis and Brignell [9] translated and validated the Problematic Social Media Use to measure levels of problematic social media use in a sample of adults aged 18–58 years, and Kokka et al. [36] translated and validated the Social Media Disorder Scale to measure levels of problematic social media use in a sample of adults aged 18–29 years. Lack of consensus among scales makes it difficult to compare results among studies and ascertain the genuine extent of the issue. Furthermore, the diverse range of instruments employed and the scarcity of a consensus concerning the cutoff points for evaluating negative social media use underscore the obstacles and difficulties associated with measuring this problem. Several scales do not even provide cutoff points, thus precluding the clear demarcation between regular and negative usage. Additionally, several scales such as the BFAS lack explicit cutoff points, opting instead to offer suggestions; in such cases, individual scholars establish different cutoff points.

Until now, most studies have used simple variables to measure TikTok usage, such as time spent, number of accounts that participants follow, and number of friends, close friends, *likes*, and *followers* [37]–[39]. Recently, two studies used an adapted version of the BFAS to measure participants' addiction to TikTok [40],[41]. In particular, these studies just replaced the term “Facebook” with “TikTok” in the six items of the BFAS. Moreover, these studies did not examine the validity of the scale referring to the TikTok items; thus, the use of the BFAS as a proxy for TikTok addiction is precarious. Additionally, Pontes et al. developed the Gaming Disorder Test (GDT) after the recognition of gaming disorder as an official behavioral addiction and mental health disorder by the World Health Organization (WHO) [42]. Authors developed four items to assess gaming activity both online and/or offline during the last 12 months. Montag and Markett introduced the TikTok Use Disorder-Questionnaire (TTUD-Q) by adapting the four GDT items [43]. In particular, they replaced the term “gaming” with “TikTok use” in the four GDT items, but they did not examine the validity of the TTUD-Q. Moreover, Montag and Markett pointed out that their study suffers from serious selection bias since they recruited their sample after an advertisement for a study on cognitive failure. Thus, they obtained a non-typical sample for TikTok users with a mean age of 41 years and a maximum age of 85 years.

Although there are plenty of tools to measure social media, social network, internet, Facebook, and Instagram addiction/disorder/problematic use, there is an absence of valid and specific psychometric tools for TikTok. Considering that the use of TikTok is increasing very rapidly and the fact that TikTok addiction may be a different form of social media addiction, there is an urge for a valid tool to measure TikTok addiction. Given the diversity in platform design among social media platforms, it is crucial to examine the impact of TikTok usage on individuals' mental health. Different platforms may have varying effects on users, potentially leading to distinct outcomes for their mental well-being. Additionally, previous research has disproportionately focused on Facebook while neglecting the popularity of TikTok and the associated concerns of maladaptive behaviors. Given the limited literature on TikTok use compared to other social media platforms, our study specifically focuses on TikTok overuse. Therefore, the aim of our study was to develop and validate a tool to measure TikTok addiction.

## 2. Materials and methods

### 2.1. Development of the scale

**Figure 1.**
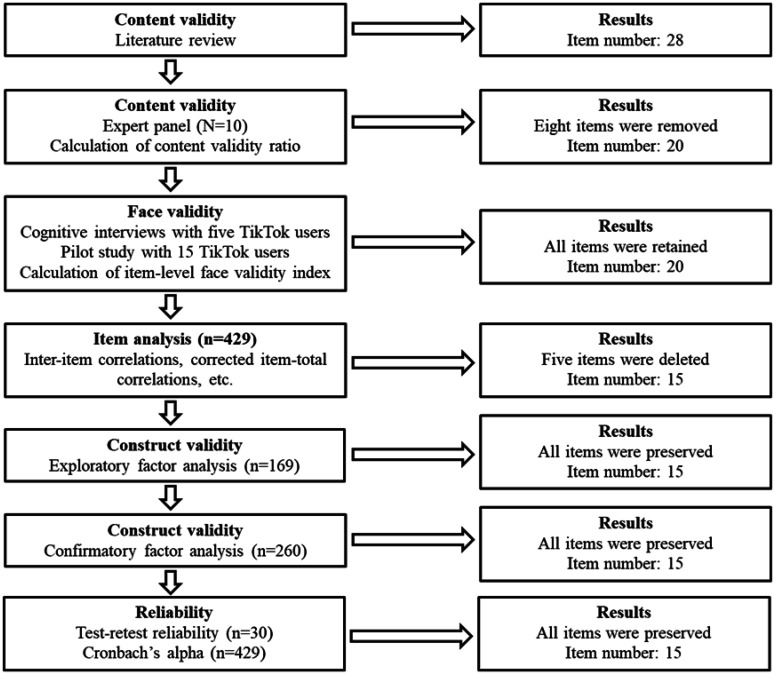
Development of the TikTok Addiction Scale.

Figure 1 shows the development and validation of the TikTok Addiction Scale (TTAS). We followed several steps to develop TTAS items [44]. First, we performed a complete and thorough literature review to identify instruments, scales, and tools that measure social media, social network, internet, Facebook, and Instagram addiction/disorder/problematic use [27]–[33],[45]–[47]. Literature suggests that addiction involves six core components: (1) salience (preoccupation with social media), (2) mood modification (social media use improves mood), (3) tolerance (increasing amounts of social media use are required to satisfy users), (4) withdrawal (users experience negative feelings when social media use is discontinued or suddenly reduced), (5) conflict (social media use causes problems and conflicts in work/education, relationships, sleep, and other activities), and (6) relapse (users revert to previous patterns of social media use after abstinence or control) [48]–[50]. In a similar way, the WHO has recently defined in the 11th Revision of the International Classification of Diseases (ICD-11) gaming disorder as a pattern of gaming behavior or otherwise digital-gaming behavior [51]. It is the first time that any type of social media addiction is defined as a disorder in the ICD. In this context, gaming disorder is characterized by loss of control, increased priority given to gaming over essential daily activities, functional impairments, and continuation of gaming despite negative consequences. Emphasis is given that symptoms of social media disorder/addiction should last for at least 12 months. Therefore, WHO's definition of gaming disorders is in accordance with the core components of addiction that we mention above. For instance, increasing priority given to gaming over essential daily activities refers to the component “conflict”. After the literature review and the identification of relevant instruments, scales, and tools, we created a pool of items that were related to the six core components of addiction. We removed items that seemed to be irrelevant to TikTok addiction. Also, we removed items with a similar meaning. Then, we matched each item to each core component of addiction. For instance, we considered the items “I think about how I could reduce my work/study time to spend more time on TikTok” and “I have TikTok in my mind even when I am not using it” to belong to the component “salience”, while items “I have had difficulties closing TikTok” and “I want to use TikTok more and more” belong to the component “tolerance”. In the end, we developed 28 items to measure TikTok addiction based on the six core components of addiction, i.e., salience (four items), mood modification (five items), tolerance (five items), withdrawal (two items), conflict (ten items), and relapse (two items).

Afterward, we employed a panel of 10 experts from different backgrounds (e.g., psychologists, mental healthcare professionals, physicians, sociologists, and nurses) to examine the content validity of the initial set of 28 items. We asked experts to rate how well each of the 28 items corresponded to TikTok addiction among users. We offered experts three options to evaluate each item: “not essential”, “useful but not essential”, or “essential”. After the experts' evaluation, we calculated the content validity ratio for each item as follows:

$$\text{Content validity ratio}=\frac{\text{n}-\frac{\text{N}}{2}}{\frac{\text{N}}{2}}$$ (1)

In the formula (1), n was the number of experts who rated an item as “essential”, while N was the total number of experts (=10). We retained items with a content validity ratio greater than 0.80, as suggested by the literature [52]. In this step, we removed eight items and, thus, 20 items remained in our scale; four items refer to salience, four items refer to mood modification, four items refer to tolerance, two items refer to withdrawal, four items refer to conflict, and two items refer to relapse.

Then, we examined the face validity of the TTAS by performing cognitive interviews with five TikTok users [53]. All users interpreted the 20 items as we intended. Furthermore, we conducted a pilot study with 15 TikTok users (eight males and seven females, mean age: 22.7 years) to examine the clarity of the 20 items by calculating the item-level face validity index. We asked TikTok users to rate the clarity of the 20 items. Answers were on a four-point Likert scale: 1=item is not clear, 2=item is somewhat clear, 3=item is quite clear, and 4=item is highly clear). Then, we calculated the item-level face validity index, and we kept items with values greater than 0.80, as suggested by the literature [54]. Face validity index ranged from 0.866 to 1.000; thus, we kept all 20 items in our scale.

Twenty items (e.g., “During the last 12 months, I felt good when I uploaded videos on TikTok”, “During the last 12 months, I thought about how I could reduce my work/study time to spend more time on TikTok”, and “During the last 12 months, I felt sad when I could not use TikTok for some time”) were rated on a five-point Likert scale as follows: very rarely (1), rarely (2), sometimes (3), often (4), very often (5). Higher scores indicate greater TikTok addiction. Supplementary Table S1 shows the 20 items that were produced after the initial development phase of the TTAS.

### 2.2. Participants and procedure

We developed the TTAS in Greek. Our study population included adults aged 18 years or older who were able to read and understand Greek. Moreover, our participants had to be TikTok users for at least the last 12 months. We created an anonymous online version of the study questionnaire through Google Forms. We collected data through several ways: dissemination through social media (i.e., TikTok, Facebook, Instagram, Viber, and WhatsApp), face-to-face interviews, and e-mail campaigns. We collected data during July 2024.

The final overall sample included 429 TikTok users. Among our participants, 81.8% were female (*n* = 351) and 18.2% were male (*n* = 78). The mean age of our sample was 26.5 years (standard deviation: 8.5), with a median value of 22 years (minimum age: 18 years; maximum age: 54 years). Participants reported a mean TikTok daily use of 2.2 hours (standard deviation: 1.6 hours; median: 2 hours; minimum value: 15 minutes; maximum value: 8 hours).

### 2.3. Item analysis

We employed an item analysis for the 20 items that were produced after the initial development phase of the TTAS. We used the overall sample to check inter-item correlations, corrected item-total correlations, floor and ceiling effects, skewness, kurtosis, and Cronbach's alpha (when a single item was deleted) for our 20 items [55]. Literature suggests that acceptable values for inter-item correlation range from 0.15 to 0.75 [56], and are higher than 0.30 for item-total correlation [57]. Floor or ceiling effects are considered when more than 85% of participants achieve the lowest or highest possible score, respectively [58]. Items follow the normal distribution when skewness is between −2 and +2, and kurtosis is between −7 and +7 [59].

### 2.4. Construct validity

We employed exploratory factor analysis (EFA) and confirmatory factor analysis (CFA) to examine the construct validity of the TTAS. Literature suggests a minimum sample size for the EFA of 50 observations [60] or five observations per item [55]. Also, the minimum sample size for the CFA is 200 observations [61]. Our sample covered these requirements. In particular, we randomly split our participants into two groups to perform EFA and CFA with different samples. Then, we used 169 TikTok users to conduct the EFA and 260 TikTok users to conduct the CFA. Therefore, we used two different samples to perform EFA and CFA to improve the validity of our analyses. Our samples in both cases covered the sample requirements for EFA and CFA.

First, we performed EFA to explore the underlying factor structure of the TTAS, and then we employed CFA to verify the results of EFA. In this step, we included the 15 items that emerged after the initial development of the TTAS and the item analysis.

We calculated the Kaiser-Meyer-Olkin index and *p*-value for the Bartlett sphericity test to examine the suitability of our data to perform EFA. Acceptable values for the Kaiser-Meyer-Olkin index and Bartlett sphericity test are >0.80 and <0.05, respectively [59]. We used oblique rotation (promax method in SPSS) to perform EFA since we expected significant correlations between potential factors that were developed from the analysis. Acceptable values for the EFA are the following: eigenvalues >1, factor loadings >0.60, communalities >0.40, and the total variance explained by the factors >65% [59]. Additionally, we calculated Cronbach's alpha for the factors that were produced by the EFA, with values > 0.7 considered to be acceptable [62].

After EFA, we performed CFA to confirm the validity of the TTAS factor structure. The TTAS followed a normal distribution and, thus, we used the maximum likelihood estimator. We checked the goodness of fit indices in CFA by calculating two indices of absolute fit [i.e., root mean square error of approximation (RMSEA) and goodness of fit index (GFI)], two indices of relative fit [i.e., normed fit index (NFI) and comparative fit index (CFI)], and one index of parsimonious fit [i.e., chi-square/degree of freedom (*χ^2^*/*df*)]. Acceptable values for fit indices in CFA are the following: RMSEA < 0.10, GFI > 0.90, NFI > 0.90, CFI > 0.90, and *χ^2^*/*df* < 5 [58],[63]–[65]. Additionally, we calculated standardized regression weights between items and factors and correlation coefficients between factors.

### 2.5. Concurrent validity

The concurrent validity of the TTAS was investigated using the Bergen Social Media Addiction Scale (BSMAS) [29], the Patient Health Questionnaire-4 (PHQ-4) [66], and the Big Five Inventory-10 (BFI-10) [67]. We checked the concurrent validity of the TTAS on the overall sample (*N* = 429).

The BSMAS is a one-dimensional scale that includes six items (e.g., “During the last 12 months, did you spend a lot of time thinking about social media or planned use of social media?” and “How often during the last year have you used social media to forget about personal problems?”). The BSMAS is a general tool that measures the levels of addiction on all social media applications that an individual uses. The BSMAS assesses problematic social media use over a 12-month period. The BSMAS measures the six core components of addiction, i.e., salience, mood modification, tolerance, withdrawal, conflict, and relapse. Answers are on a five-point Likert scale: from 1 (very rarely) to 5 (very often). Total score ranges from 6 to 30, and higher scores on the BSMAS indicate greater social media addiction. The BSMAS has been translated and validated in several languages such as Spanish, French, German, Swedish, Polish, Slovene, Romanian, Russian, Korean, and Chinese [68]–[72]. We used the valid Greek version of the BSMAS [34]. In our study, Cronbach's alpha for the BSMAS was 0.829, and McDonald's Omega was 0.830.

Two recent systematic reviews suggested a correlation between social media overuse and depression and anxiety symptoms [73],[74]. Thus, we used the PHQ-4 to further examine the concurrent validity of the TTAS. The PHQ-4 comprises four items (e.g., “Over the last two weeks, how often were you bothered by feeling nervous, anxious, or on edge?”) to assess anxiety and depression. In particular, two items measure anxiety (i.e., “Over the last two weeks, how often have you been bothered by feeling nervous, anxious, or on edge?” and “Over the last two weeks, how often were you not able to stop or control worrying?”) and two items measure depression (i.e., “Over the last two weeks, how often have you felt little interest or pleasure in doing things?” and “Over the last two weeks, how often did you feel down, depressed, or hopeless?”). Answers are on a four-point Likert scale, from 0 (not at all) to 3 (nearly every day). Total score ranges from 0 to 12, and higher scores on the PHQ-4 indicate greater anxiety and depression. A recent systematic review identified 26 studies from 19 countries and found that the PHQ-4 has great psychometric properties (reliability and validity) for clinical and nonclinical populations [75]. The PHQ-4 has been translated and validated in more than 20 languages (e.g., German, Swedish, Austrian, Portuguese, Croatian, Georgian, Japanese, and Chinese) with different sample populations (e.g., general population, patients, young adults, students, pregnant women, and athletes) [75]–[78]. We used the valid Greek version of the PHQ-4 [79]. In our study, Cronbach's alpha for the PHQ-4 was 0.818, and McDonald's Omega was 0.825.

Several studies support a positive correlation between social media addiction and neuroticism [80]–[82]. Moreover, literature has found a negative correlation between social media addiction and conscientiousness [82]. Therefore, we used the BFI-10 [67] to examine the concurrent validity of the TTAS. The BFI-10 is a 10-item scale that measures the domains of the five-factor model of personality: neuroticism, extraversion, openness, agreeableness, and conscientiousness. Each factor includes two items. A sample item is the following: “I see myself as someone who is reserved”. Answers are on a five-point Likert scale from 1 (strongly disagree) to 5 (strongly agree). The BFI-10 evaluates the main personality dimensions. Total score for each factor ranges from 2 to 10. Higher scores on the BFI-10 indicate greater neuroticism, extraversion, openness, agreeableness, and conscientiousness. The BFI-10 has been validated in several languages such as English, German, Indian, Brazilian, and Romanian [67],[83]–[85]. We used the valid Greek version of the BFI-10 [86]. In our study, Cronbach's alpha for BFI-10 was 0.712, and McDonald's Omega was 0.714.

We measured the overall score for the scale and the six factors that emerged from the factor analysis. Specifically, we summed the responses for all items and divided the total by the number of items to determine the total score for the scale. Likewise, scores for each factor were calculated. All the scores ranged from 1 to 5, with higher scores indicating greater levels of TikTok addiction.

We expected a positive correlation between the TTAS and the BSMAS, the PHQ-4, and neuroticism. Moreover, we expected a negative correlation between the TTAS and extraversion, openness, and conscientiousness.

### 2.6. Reliability

First, we used the overall sample (*N* = 429) to assess the reliability of the TTAS. In particular, we calculated Cronbach's alpha and McDonald's Omega for the TTAS and the factors. Acceptable values for Cronbach's alpha and McDonald's Omega are >0.6 [62].

Additionally, we measured corrected item-total correlations and Cronbach's alpha when a single item was deleted for the 15 items of the TTAS. Acceptable values for corrected item-total correlations were ≥0.30 [57].

Moreover, we performed a test-retest study with 30 TikTok users. In that case, participants completed the TTAS twice in one week. We measured Cohen's kappa for the 15 items of the TTAS since the answers were on an ordinal scale. Also, we measured the two-way mixed intraclass correlation coefficient (absolute agreement) for the total score of TTAS and for scores on six factors.

### 2.7. Ethical considerations

We collected our data on an anonymous and voluntary basis. We informed participants about the aim and the design of our study, and they gave their informed consent. The Ethics Committee of the Faculty of Nursing, National and Kapodistrian University of Athens approved our study protocol (approval number; 451, June 2023). Moreover, we conducted our study in accordance with the Declaration of Helsinki [87].

### 2.8. Statistical analysis

We use absolute numbers and percentages to present categorical variables. Also, we use mean, standard deviation, median, minimum value, and maximum value to present continuous variables. We employed the Kolmogorov-Smirnov test and Q-Q plots to examine the distribution of scores on our scales. Scores on TTAS, BSMAS, PHQ-4, and BFI-10 followed normal distribution. Thus, we calculated Pearson's correlation coefficient to examine the correlation between scales. *P*-values less than 0.05 were considered statistically significant. We performed CFA with AMOS version 21 (Amos Development Corporation, 2018). All other analyses were conducted with IBM SPSS 21.0 (IBM Corp. Released 2012. IBM SPSS Statistics for Windows, Version 21.0. Armonk, NY: IBM Corp.).

## 3. Results

### 3.1. Item analysis

We present results from the item analysis in Table 1: descriptive statistics, corrected item-total correlations, floor and ceiling effects, skewness, kurtosis, and Cronbach's alpha when a single item was deleted. Moreover, we present inter-item correlations between the 20 items that were produced after the initial development phase of the TikTok Addiction Scale in Supplementary Table S2.

We deleted items #1, #4, #5, #6, and #12 due to low inter-item correlations with several other items. Moreover, items #1 and #5 had negative inter-item correlations with other items, while item #5 had low corrected item-total correlation (0.268). Cronbach's alpha for the 20 items was 0.896 and decreased after the removal of each single item. Moreover, we examined the meaning of retained and excluded items in a theoretical way to judge the validity of item analysis. All items that were removed after item analysis had similar meanings that were retained. For instance, item #1 had a similar meaning as item #2, item #4 as item #3, item #5 as item #7, item #6 as item #8, and item #12 as item #11.

Therefore, we deleted five items (#1, #4, #5, #6, #12; Table 1); the remaining 15 items had acceptable corrected item-total correlations, inter-item correlations, floor and ceiling effects, skewness, and kurtosis. Cronbach's alpha for the 15 items was 0.916.

**Table 1.** Descriptive statistics, corrected item-total correlations, floor and ceiling effects, skewness, kurtosis, and Cronbach's alpha (when a single item was deleted) for the 20 items that were produced after the initial development phase of the TikTok Addiction Scale (*N* = 429).

**Item**	**Mean (standard deviation)**	**Corrected item-total correlation**	**Floor effect (%)**	**Ceiling effect (%)**	**Skewness**	**Kurtosis**	**Cronbach's alpha if item deleted**	**Item exclusion or retention**
1. I spend a lot of time thinking about which videos should I upload on TikTok	1.42 (0.77)	0.316	73.0	0.0	1.76	2.12	0.889	Excluded
2. I think about how I could reduce my work/study time to spend more time on TikTok	1.57 (0.79)	0.520	58.7	0.2	1.33	1.32	0.884	Retained
3. I have TikTok in my mind even when I am not using it	1.94 (0.98)	0.628	41.3	1.9	0.87	0.24	0.881	Retained
4. I feel compelled to upload videos on TikTok soon after an event	1.35 (0.66)	0.350	74.8	0.0	1.93	3.08	0.888	Excluded
5. I feel good when I upload videos on TikTok	1.77 (1.07)	0.268	60.1	1.4	1.11	0.04	0.892	Excluded
6. My mood is improved when I get likes/comments for my videos	2.02 (1.30)	0.321	54.5	5.1	0.88	−0.61	0.892	Excluded
7. I feel calm when I use TikTok	3.27 (0.96)	0.333	5.8	6.3	−0.53	0.04	0.889	Retained
8. I use TikTok as a getaway from my problems and my thoughts	3.43 (1.16)	0.549	6.8	20.7	−0.35	−0.61	0.883	Retained
9. I have had difficulties controlling the time I spend on TikTok	3.27 (1.22)	0.662	9.1	19.1	−0.19	−0.87	0.879	Retained
10. I have had difficulties closing TikTok	3.00 (1.19)	0.641	12.4	12.8	0.01	−0.78	0.880	Retained
11. I want to use TikTok more and more	2.36 (1.08)	0.593	23.8	5.1	0.57	−0.12	0.881	Retained
12. I use TikTok even in the bathroom	3.08 (1.38)	0.347	17.2	20.0	−0.09	−1.24	0.891	Excluded
13. I feel bad when I cannot use TikTok for some time	1.34 (0.64)	0.590	73.4	0.0	2.05	4.24	0.884	Retained
14. I feel sad when I cannot use TikTok for some time	1.30 (0.55)	0.574	75.1	0,0	1.71	1.96	0.885	Retained
15. I don't get enough time to do things I want to do because I spend a lot of time on TikTok	1.98 (1.06)	0.603	42.4	2.8	0.90	0.14	0.881	Retained
16. I lose sleep due to excessive use of TikTok	2.47 (1.14)	0.614	23.8	5.8	0.41	−0.53	0.881	Retained
17. I am not able to concentrate on my work/study due to TikTok use	2.20 (1.19)	0.691	35.9	5.6	0.76	−0.32	0.878	Retained
18. I use TikTok so much that it has had a negative impact on my work/study	1.97 (1.16)	0.627	47.6	4.4	1.04	0.16	0.880	Retained
19. I feel depressed when I do not use TikTok, which disappears when I use it	1.63 (0.89)	0.534	59.7	0.7	1.33	1.09	0.884	Retained
20. I feel anxious when I do not use TikTok, which disappears when I use it	1.70 (0.94)	0.577	57.6	0.5	1.13	0.27	0.882	Retained

### 3.2. Exploratory factor analysis

The Kaiser-Meyer-Olkin index was 0.895 and the *p*-value for Bartlett sphericity was <0.001, indicating that our sample was adequate to perform EFA. We employed oblique rotation (promax method) to perform our EFA, including the 15 items mentioned above (items #2, #3, #7, #8, #9, #10, #11, #13, #14, #15, #16, #17, #18, #19, and #20 in Table 1).

We found six factors including all items (Table 2). Therefore, our EFA confirmed the six-factor model for the TTAS that we hypothesized in the Introduction. According to the literature, addiction involves salience, mood modification, tolerance, withdrawal, conflict, and relapse. Thus, our EFA identified the following factors: salience (two items, #1, #2), mood modification (two items, #3, #4), tolerance (three items, #5, #6, #7), withdrawal symptoms (two items, #8, #9), conflict (four items, #10, #11, #12, #13), and relapse (two items, #14, #15) (Table 2). The total variance explained by the six factors was 80.703%. The variance explained by each single factor was as follows: 47.649% of the total variance was explained by the factor “conflict”, 9.605% of the total variance was explained by the factor “tolerance”, 8.559% of the total variance was explained by the factor “withdrawal symptoms”, 5.823% of the total variance was explained by the factor “salience”, 4.908% of the total variance was explained by the factor “relapse”, and 4.159% of the total variance was explained by the factor “mood modification”. Factor loadings ranged from 0.733 to 0.939, while communalities ranged from 0.707 to 0.894.

Cronbach's alpha for the TTAS was 0.916, while McDonald's Omega was 0.923. Cronbach's alpha and McDonald's Omega for the factors of the TTAS ranged from 0.659 to 0.868 (Supplementary Table S3).

### 3.3. Confirmatory factor analysis

**Figure 2.**
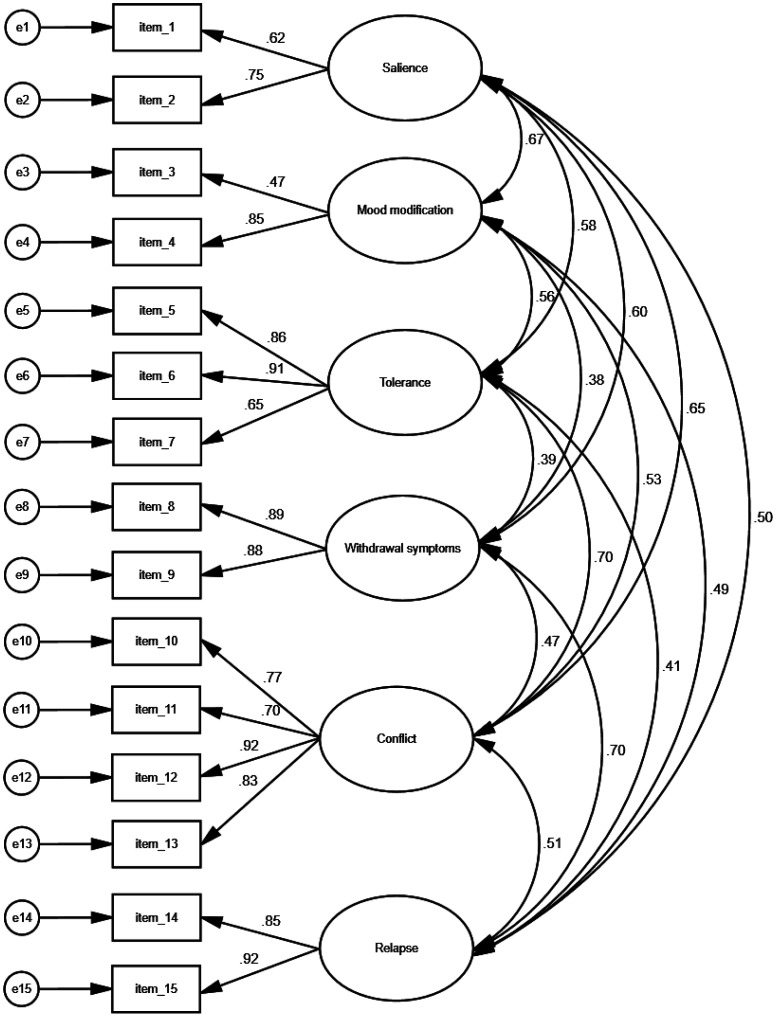
Confirmatory factor analysis of the TikTok Addiction Scale.

Then, we performed CFA to verify the factors of the TTAS that were obtained from the EFA. Thus, we performed CFA of 15 items across six factors. Our CFA suggested that the six-factor model with 15 items of the TTAS had a very good fit to data since *χ^2^*/*df* was 1.481, RMSEA was 0.043, GFI was 0.953, NFI was 0.953, and CFI was 0.984. Moreover, the correlation coefficients between factors were positive and statistically significant (*p* < 0.001 in all cases). In particular, correlation coefficients ranged from 0.382 to 0.703. Standardized regression weights between 15 items and six factors ranged from 0.473 to 0.921 (*p* < 0.001 in all cases). CFA of the TTAS is shown in Figure 2.

In conclusion, our EFA and CFA identified a six-factor 15-item model for the TTAS: salience (two items, #1, #2), mood modification (two items, #3, #4), tolerance (three items, #5, #6, #7), withdrawal symptoms (two items, #8, #9), conflict (four items, #10, #11, #12, #13), and relapse (two items, #14, #15) (Supplementary Table S4).

**Table 2.** Exploratory factor analysis using oblique rotation (promax method) for the TikTok Addiction Scale (*n* = 169).

**Item**	**Factors**						**Communalities**
	**Conflict**	**Tolerance**	**Withdrawal symptoms**	**Salience**	**Relapse**	**Mood modification**	
1. I think about how I could reduce my work/study time to spend more time on TikTok	0.391	0.350	0.443	0.866	0.344	0.224	0.770
2. I have TikTok in my mind even when I am not using it	0.599	0.528	0.555	0.796	0.358	0.220	0.707
3. I feel calm when I use TikTok	0.196	0.288	0.191	0.075	0.370	0.903	0.845
4. I use TikTok as a getaway from my problems and my thoughts	0.471	0.422	0.395	0.492	0.318	0.809	0.788
5. I have had difficulties controlling the time I spend on TikTok	0.667	0.869	0.227	0.339	0.398	0.430	0.809
6. I have had difficulties closing TikTok	0.699	0.888	0.427	0.499	0.306	0.266	0.843
7. I want to use TikTok more and more	0.527	0.883	0.489	0.378	0.434	0.285	0.824
8. I feel bad when I cannot use TikTok for some time	0.403	0.408	0.898	0.533	0.464	0.220	0.810
9. I feel sad when I cannot use TikTok for some time	0.497	0.430	0.894	0.445	0.482	0.317	0.836
10. I don't get enough time to do things I want to do because I spend a lot of time on TikTok	0.829	0.592	0.442	0.488	0.439	0.059	0.767
11. I lose sleep due to excessive use of TikTok	0.733	0.664	0.220	0.105	0.485	0.423	0.722
12. I am not able to concentrate on my work/study due to TikTok use	0.917	0.663	0.392	0.449	0.486	0.324	0.847
13. I use TikTok so much that it has had a negative impact on my work/study	0.876	0.528	0.474	0.487	0.355	0.307	0.804
14. I feel depressed when I do not use TikTok, which disappears when I use it	0.503	0.421	0.430	0.317	0.939	0.403	0.894
15. I feel anxious when I do not use TikTok, which disappears when I use it	0.467	0.464	0.631	0.510	0.867	0.294	0.841

Note: Values express factors loadings. Bold indicates the highest factor loadings for the items.

### 3.4. Concurrent validity

We found a positive correlation between the TTAS and the BSMAS, suggesting that participants with higher levels of social media addiction also have higher levels of TikTok addiction. In particular, correlation coefficients between the TTAS and the BSMAS ranged from 0.475 to 0.744 (*p* < 0.01 in all cases). Moreover, we found a positive correlation between the TTAS and the PHQ-4, suggesting that participants with higher levels of anxiety and depression may have higher levels of TikTok addiction. In particular, correlation coefficients between the TTAS and the PHQ-4 ranged from 0.163 to 0.371 (*p* < 0.01 in all cases).

Additionally, the six factors of the TTAS and the total score were correlated negatively with conscientiousness; correlation coefficients ranged from −0.406 to −0.210 (*p* < 0.01 in all cases). Similarly, we found a negative correlation between the extraversion and salience (*r* = −0.123, *p* < 0.05), mood modification (*r* = −0.226, *p* < 0.01), tolerance (*r* = −0.110, *p* < 0.05), conflict (*r* = −0.165, *p* < 0.05), relapse (*r* = −0.144, *p* < 0.01), and total score of the TTAS (*r* = −0.184, *p* < 0.01).

Also, we found a negative correlation between openness and withdrawal symptoms (*r* = −0.145, *p* < 0.01) and a positive correlation between neuroticism and mood modification (*r* = 0.116, *p* < 0.05) and tolerance (*r* = 0.169, *p* < 0.01).

Therefore, the concurrent validity of the TTAS was excellent. Table 3 shows the correlations between the TTAS and the BSMAS, the PHQ-4, and the BFI-10.

**Table 3.** Pearson's correlation coefficients between the TikTok Addiction Scale (TTAS) and the Bergen Social Media Addiction Scale (BSMAS), the Patient Health Questionnaire-4 (PHQ-4), and the Big Five Inventory-10 (BFI-10) (*n* = 429).

**TTAS**	**BSMAS**	**PHQ-4**	**BFI-10**			
			**Neuroticism**	**Openness**	**Extraversion**	**Conscientiousness**
Salience	0.509**	0.163**	−0.014	−0.079	−0.123*	−0.264**
Mood modification	0.475**	0.363**	0.116*	−0.059	−0.226**	−0.210**
Tolerance	0.601**	0.317**	0.169**	−0.004	−0.110*	−0.312**
Withdrawal symptoms	0.509**	0.181**	−0.047	−0.145**	−0.037	−0.270**
Conflict	0.649**	0.358**	0.063	−0.073	−0.165*	−0.420**
Relapse	0.547**	0.266**	0.001	−0.091	−0.144**	−0.248**
Total score	0.744**	0.371**	0.089	−0.083	−0.184**	−0.406**

Note: * *p* < 0.05; ** *p* < 0.01.

### 3.5. Reliability

We present Cronbach's alpha and McDonald's Omega for the six-factor model with 15 items for the TTAS in Table 4. Cronbach's alpha and McDonald's Omega for the TTAS were 0.911 and 0.914, respectively. Moreover, Cronbach's alpha for the six factors ranged from 0.624 to 0.860, while McDonald's Omega ranged from 0.862 to 0.879. Thus, the internal consistency of the TTAS was very good.

Additionally, corrected item-total correlations had values between 0.333 and 0.756, while removal of each single item did not increase Cronbach's alpha (Supplementary Table S5).

Cohen's kappa for the 15 items ranged from 0.760 to 0.954 (*p* < 0.001 in all cases), (Supplementary Table S6). Additionally, the intra-class correlation coefficient for the total score was 0.994 (95% confidence interval: 0.984 to 0.998, *p* < 0.001), while for the six factors it ranged from 0.930 to 0.992 (*p* < 0.001 in all cases) (Supplementary Table S7). Thus, the reliability of the TTAS was excellent.

**Table 4.** Cronbach's alpha and McDonald's Omega for the six-factor model with 15 items for the TikTok Addiction Scale (*n* = 429).

**Factor**	**Cronbach's alpha**	**McDonald's Omega**
Salience	0.642	NC
Mood modification	0.624	NC
Tolerance	0.850	0.862
Withdrawal symptoms	0.847	NC
Conflict	0.873	0.879
Relapse	0.860	NC
TikTok Addiction Scale	0.911	0.914

Note: NC: non-computable due to limited items.

## 4. Discussion

Our study developed and validated a novel scale to measure levels of TikTok addiction among users. We employed a convenience sample of 429 adults in Greece to develop and evaluate the psychometric properties of TTAS. The TTAS is proven to be a reliable and valid scale to measure problematic TikTok use. The TTAS is a six-factor 15-item scale that individuals can answer in a few minutes. In particular, factor analysis revealed a six-factor structure, i.e., salience, mood modification, tolerance, withdrawal symptoms, conflict, and relapse.

Only seven years after its initial release, TikTok has become one of the world's most widely used applications for short-form videos, with more than 20% of adults around the world using it [13]. Although research on social media addiction has primarily focused on well-established platforms like Facebook, Instagram, and others, it has overlooked the influence of TikTok and related maladaptive behaviors [17]. Therefore, employing valid tools to assess TikTok addiction is essential to identify high-risk individuals. A recent review revealed that there are 37 instruments that measure negative social networking site usage, such as the Bergen Facebook Addiction Scale, the Social Media Disorder Scale, the Facebook Intrusion Questionnaire, the Generalized Problematic Internet Use Scale, and the Internet Addiction Test [27]. However, no valid and specific psychometric tools exist to assess TikTok addiction/disorder/problematic use. Given the rapid increase in TikTok usage and the fact that TikTok addiction may be a distinct form of social media addiction, there is a need for a valid tool to measure TikTok addiction. Given the differences in platform design among social media platforms, it is crucial to investigate the impact of TikTok usage on individuals' mental health. To the best of our knowledge, no valid and specific tool exists to assess TikTok addiction.

Although there are several instruments to measure problematic social media usage, there is no one to specifically measure problematic TikTok usage. Since TikTok seems to be more addictive than other social media applications, there is an urgent need to measure TikTok addiction in a valid way with specific scales. Moreover, the different way that TikTok works requires a valid measurement with a scale with robust psychometric properties. In particular, TikTok's functionality is rooted in the psychological concept of intermittent reinforcement, as its endless stream of videos is inherently addictive. Users are constantly anticipating a reward, such as an amusing clip, which triggers a dopamine release in their brains [21]–[24]. Moreover, the increased dopaminergic activity resulting from receiving a *like* promotes continued TikTok usage and content creation, as users seek to replicate the pleasurable experience. The *like* feature provides gratification in multiple ways: users enjoy both receiving and giving likes, similar to the satisfaction derived from gift-giving. In both instances, the immediate gratification facilitated by the like button fosters habitual use and dependency through positive reinforcement [25],[26].

In this context, we developed and validated a specific tool to measure TikTok addiction among users, i.e., the TikTok Addiction Scale. Since the literature suggests that addiction involves six core components, namely salience, mood modification, tolerance, withdrawal, conflict, and relapse, we developed items for the TTAS according to this theoretical framework [48]–[51]. After a thorough literature review [27]–[33],[45]–[47], we identified 28 items and deleted 8 after examination of content validity and face validity. Then, we performed an item analysis for the 20 items that were produced after the initial development phase of the TTAS and deleted 5 items due to low inter-item correlations, negative inter-item correlations, and low corrected item-total correlations.

Afterward, we performed exploratory and confirmatory factor analysis to examine the construct validity of the TTAS. Factor analysis identified six factors that explained 80.703% of the total variance of TikTok addiction. The factors were the following: salience (two items), mood modification (two items), tolerance (three items), withdrawal symptoms (two items), conflict (four items), and relapse (two items). The factor “conflict” explained the greatest amount of the variance, and the factors “tolerance” and “withdrawal symptoms” followed. In CFA, the RMSEA was 0.043, the GFI was 0.953, the NFI was 0.953, and the CFI was 0.984, which indicates a very good fit to our data [58],[63]–[65]. Additionally, the correlation coefficients between the six factors ranged from 0.382 to 0.703 and were statistically significant (*p* < 0.001 in all cases). Thus, our factor analysis identified a six-factor 15-item model for the TTAS and confirmed our hypothesis that TikTok addiction involves six components (i.e., salience, mood modification, tolerance, withdrawal, conflict, and relapse) as other addictions involve.

Additionally, we examined the concurrent validity of the TTAS by estimating the correlation between the TTAS and the BSMAS [29], the PHQ-4 [66], and the BFI-10 [67]. In particular, we expected a positive correlation between the TTAS and the BSMAS, the PHQ-4, and neuroticism. On the opposite, we expected a negative correlation between the TTAS and extraversion, openness, and conscientiousness. The concurrent validity of the TTAS was excellent since we found moderate to high correlation coefficients between the TTAS and the BSMAS. As expected, the scores for the TTAS were correlated with a specific social media measure of addiction, such as the Bergen Social Media Addiction Scale. Moreover, we found positive and statistically significant correlations between the TTAS and the PHQ-4. Literature confirms this finding since two recent systematic reviews found a positive correlation between social media overuse and depression and anxiety symptoms [73],[74]. Also, we found that the higher the score on the TTAS, the higher the score on neuroticism. Several studies showed a positive correlation between social media addiction and neuroticism [80]–[82]. On the other hand, we found a negative correlation between the TTAS and conscientiousness. This finding is in accordance with the literature since individuals with high levels of conscientiousness give less priority to social media in order to accomplish their work [82].

Finally, we found that the reliability of the TTAS was excellent since Cronbach's alpha and McDonald's Omega for the scale were 0.911 and 0.914, respectively. Moreover, Cronbach's alpha for the six factors ranged from 0.624 to 0.860, while McDonald's Omega ranged from 0.862 to 0.879. Additionally, in the test-retest study, we found that the intraclass correlation coefficient for the TTAS was 0.994, while for the six factors it ranged from 0.930 to 0.992.

Our study had several limitations. First, we conducted our study in a particular country (i.e., Greece) by employing a convenience sample of 429 adults aged 18–54 years. For instance, compared with the general population, a relatively low percentage of males participated in our study. Although our sample included females and males with an age range from 18 to 54 years, it cannot be considered representative of the general population. Thus, our sample cannot be generalized, and further studies with more representative samples and in different settings (e.g., students and adolescents) should be conducted to further examine the validity of the TTAS. Further studies should be conducted in different cultural contexts to provide data for cross-cultural validation. However, our psychometric analysis is powerful since our sample size met all the requirements. Second, we did not perform our study in clinical settings, and, thus, our findings should be used with concern in clinical practice for diagnosis. Studies with well-controlled clinical settings would add significant information. For instance, identification of TikTok-addicted users through clinical examination by psychologists may help us to identify cutoff points for the TTAS and examine the predictive validity of the scale. Third, we used self-report scales to measure the concurrent validity of the TTAS, and, thus, information bias is probable. Fourth, we examined the concurrent validity of the TTAS by calculating the correlation coefficients between the TTAS and three other scales, i.e., BSMAS, PHQ-4, and BFI-10. Future studies may also use other scales to further validate the TTAS. Fifth, we used a convenience sample collecting data through social media, face-to-face interviews, and e-mail campaigns, and, thus, selection bias is probable. For instance, older people use social media and e-mail less often and may be under-represented in our sample. Additionally, we cannot estimate the response rate since we collected our data through social media and e-mail campaigns. Finally, we employed a cross-sectional design to examine the validity of the TTAS. Since TikTok users' attitudes may change over time, longitudinal studies should investigate how TikTok addiction levels change over time. Longitudinal studies could provide information on the stability and predictive validity of the TTAS by measuring changes in levels of TikTok addiction over time.

## 5. Conclusions

As far as we are aware, the TTAS is the first tool to specifically measure levels of TikTok addiction among users. After a thorough reliability and validity analysis, we found that the TTAS is a short and easy-to-use tool with robust psychometric properties. Our findings suggest that the TTAS is a six-factor 15-item scale that measures the six core components of addiction (i.e., salience, mood modification, tolerance, withdrawal, conflict, and relapse). Thus, the TTAS may be used as a timely tool to measure levels of TikTok addiction and identify high-risk users, both in the community and educational fields. Considering the limitations of our study, we recommend the translation and validation of the TTAS in other languages and populations to further examine the reliability and validity of the scale. The TTAS could be an effective means to measure TikTok addiction and may help policymakers, health educators, clinicians, and scholars to recognize high-risk groups for TikTok addiction.

## Use of AI tools declaration

The authors declare they have not used Artificial Intelligence (AI) tools in the creation of this article.


