# Supplementary material for: The TikTok Addiction Scale: Development and validation
Source: AIMS Public Health. 2024 Dec 11;11(4):1172–97. doi: 10.3934/publichealth.2024061 (PMC11717542; doi:10.3934/publichealth.2024061)
Supplement: Supplementary file 1 [file publichealth-11-04-061-s001.pdf]

Public Health

AIMS Public Health, 11(4): 1172–1197.
DOI: 10.3934/publichealth.2024061
Received: 24 September 2024
Revised: 08 November 2024
Accepted: 09 December 2024
Published: 11 December 2024

https://www.aimspress.com/journal/aimsph

Research article

The TikTok Addiction Scale: Development and validation

Petros Galanis[1,*], Aglaia Katsiroumpa[1], Ioannis Moisoglou[2] and Olympia Konstantakopoulou[1]

[1] Clinical Epidemiology Laboratory, Faculty of Nursing, National and Kapodistrian University of Athens, Athens, Greece
[2] Faculty of Nursing, University of Thessaly, Larissa, Greece

* **Correspondence:** Email: pegalan@nurs.uoa.gr; Tel: +30002107461471.

Supplementary

Table S1. The 20 items that were produced after the initial development phase of the TikTok Addiction Scale.

During the last 12 months …	**Answers**					**Factor**
	**Very rarely**	**Rarely**	**Sometimes**	**Often**	**Very often**	
1. I spend a lot of time thinking about what videos should upload on TikTok						Salience
2. I think about how I could reduce my work/study to spend more time on TikTok						Salience
3. I have TikTok in my mind even when I am not using it						Salience
4. I feel compelled to upload videos on TikTok soon after an event						Salience
5. I feel good when I upload videos on TikTok						Mood modification
6. My mood is improved when I get likes/comments for my videos						Mood modification
7. I feel calm when I use TikTok						Mood modification
8. I use TikTok as a get away from my problems and my thoughts						Mood modification
9. I have had difficulties controlling the time I spend on TikTok						Tolerance
10. I have had difficulties closing TikTok						Tolerance
11. I want to use TikTok more and more						Tolerance
12. I use TikTok even in the bathroom						Tolerance
13. I feel bad when I cannot use TikTok for some time						Withdrawal symptoms
14. I feel sad when I cannot use TikTok for some time						Withdrawal symptoms
15. I don’t get enough time to do things I want to do because I spend a lot of time on TikTok						Conflict
16. I lose sleep due to excessive use of TikTok						Conflict
17. I am not able to concentrate on my work/study due to TikTok use						Conflict
18. I use TikTok so much that it has had a negative impact on my work/study						Conflict
19. I feel depressed when I do not use TikTok, which goes when I use it						Relapse
20. I feel anxious when I do not use TikTok, which goes when I use it						Relapse

Table S2. Inter-item correlations between the 20 items that were produced after the initial development phase of the TikTok Addiction Scale (*N* = 429).

**Item**	**2**	**3**	**4**	**5**	**6**	**7**	**8**	**9**	**10**	**11**	**12**	**13**	**14**	**15**	**16**	**17**	**18**	**19**	**20**
**1**	0.326	0.197	0.596	0.560	0.625	0.039	0.063	0.174	0.111	0.208	-0.016	0.208	0.154	0.063	0.102	0.046	0.017	0.110	0.135
**2**		0.484	0.358	0.224	0.279	0.133	0.365	0.265	0.305	0.334	0.154	0.404	0.408	0.320	0.234	0.340	0.347	0.307	0.334
**3**			0.296	0.212	0.229	0.185	0.428	0.403	0.480	0.380	0.263	0.421	0.403	0.491	0.334	0.502	0.462	0.317	0.406
**4**				0.626	0.585	0.081	0.165	0.050	0.076	0.193	0.065	0.325	0.235	0.085	0.029	0.037	0.078	0.088	0.163
**5**					0.761	0.030	0.063	0.050	-0.025	0.076	0.129	0.250	0.227	-0.014	0.031	-0.009	0.015	0.036	0.095
**6**						0.072	0.099	0.155	0.054	0.157	0.118	0.243	0.184	0.007	0.147	0.051	0.013	0.117	0.095
**7**							0.462	0.280	0.194	0.193	0.241	0.178	0.200	0.120	0.255	0.205	0.163	0.300	0.309
**8**								0.429	0.425	0.363	0.265	0.303	0.337	0.298	0.366	0.410	0.415	0.365	0.379
**9**									0.762	0.554	0.324	0.300	0.272	0.502	0.585	0.582	0.495	0.337	0.361
**10**										0.644	0.261	0.334	0.331	0.560	0.496	0.593	0.509	0.288	0.354
**11**											0.191	0.402	0.441	0.404	0.415	0.457	0.338	0.335	0.410
**12**												0.158	0.147	0.233	0.360	0.343	0.282	0.069	0.095
**13**													0.742	0.375	0.290	0.385	0.419	0.471	0.581
**14**														0.355	0.294	0.381	0.393	0.505	0.559
**15**															0.537	0.701	0.613	0.393	0.406
**16**																0.631	0.540	0.406	0.353
**17**																	0.767	0.436	0.400
**18**																		0.421	0.432
**19**																			0.756
**20**																			

Table S3. Cronbach's alpha and McDonald's Omega for the six-factor model for the TikTok Addiction Scale that developed after the exploratory factor analysis (n = 169).

Factor	Cronbach's alpha	McDonald's Omega
Salience	0.659	NC
Mood modification	0.683	NC
Tolerance	0.865	0.871
Withdrawal symptoms	0.794	NC
Conflict	0.868	0.876
Relapse	0.836	NC
TikTok Addiction Scale	0.916	0.923

Note: NC: non computable due to limited items.

Table S4. The six-factor 15-item model for the TikTok Addiction Scale.

During the last 12 months …	Answers				
	Very rarely	Rarely	Sometimes	Often	Very often
1. I think about how I could reduce my work/study to spend more time on TikTok					
2. I have TikTok in my mind even when I am not using it					
3. I feel calm when I use TikTok					
4. I use TikTok as a get away from my problems and my thoughts					
5. I have had difficulties controlling the time I spend on TikTok					
6. I have had difficulties closing TikTok					
7. I want to use TikTok more and more					
8. I feel bad when I cannot use TikTok for some time					
9. I feel sad when I cannot use TikTok for some time					
10. I don't get enough time to do things I want to do because I spend a lot of time on TikTok					
11. I lose sleep due to excessive use of TikTok					
12. I am not able to concentrate on my work/study due to TikTok use					
13. I use TikTok so much that it has had a negative impact on my work/study					
14. I feel depressed when I do not use TikTok, which goes when I use it					
15. I feel anxious when I do not use TikTok, which goes when I use it					

Six factors:

- Salience (two items; #1, #2)
- Mood modification (two items; #3, #4)
- Tolerance (three items; #5, #6, #7)
- Withdrawal symptoms (two items; #8, #9)
- Conflict (four items; #10, #11, #12, #13)
- Relapse (two items; #14, #15)

Sum the answers in all items and divide the aggregate by total number of answers in order to calculate the total score for the TikTok Addiction Scale. Similarly, calculate the score for each factor.

All scores range from 1 to 5. Higher scores indicate higher levels of TikTok Addiction.

Table S5. Corrected item-total correlations and Cronbach's alpha (when a single item was deleted) for the six-factor model with 15 items for the TikTok Addiction Scale (*n* = 429).

Items	Corrected item-total correlations	Cronbach's alpha (when a single item was deleted)
1. I think about how I could reduce my work/study to spend more time on TikTok	0.471	0.906
2. I have TikTok in my mind even when I am not using it	0.605	0.451
3. I feel calm when I use TikTok	0.333	0.911
4. I use TikTok as a get away from my problems and my thoughts	0.569	0.904
5. I have had difficulties controlling the time I spend on TikTok	0.687	0.899
6. I have had difficulties closing TikTok	0.701	0.898
7. I want to use TikTok more and more	0.608	0.902
8. I feel bad when I cannot use TikTok for some time	0.567	0.904
9. I feel sad when I cannot use TikTok for some time	0.571	0.905
10. I don't get enough time to do things I want to do because I spend a lot of time on TikTok	0.667	0.900
11. I lose sleep due to excessive use of TikTok	0.635	0.901
12. I am not able to concentrate on my work/study due to TikTok use	0.756	0.896
13. I use TikTok so much that it has had a negative impact on my work/study	0.691	0.899
14. I feel depressed when I do not use TikTok, which goes when I use it	0.583	0.903
15. I feel anxious when I do not use TikTok, which goes when I use it	0.619	0.902

Table S6. Cohen's kappa for the six-factor model with 15 items for the TikTok Addiction Scale (*n* = 30).

Items	Cohen's kappa	*p*-value
1. I think about how I could reduce my work/study to spend more time on TikTok	0.811	<0.001
2. I have TikTok in my mind even when I am not using it	0.818	<0.001
3. I feel calm when I use TikTok	0.811	<0.001
4. I use TikTok as a get away from my problems and my thoughts	0.868	<0.001
5. I have had difficulties controlling the time I spend on TikTok	0.913	<0.001
6. I have had difficulties closing TikTok	0.912	<0.001
7. I want to use TikTok more and more	0.951	<0.001
8. I feel bad when I cannot use TikTok for some time	0.814	<0.001
9. I feel sad when I cannot use TikTok for some time	0.760	<0.001
10. I don't get enough time to do things I want to do because I spend a lot of time on TikTok	0.950	<0.001
11. I lose sleep due to excessive use of TikTok	0.954	<0.001
12. I am not able to concentrate on my work/study due to TikTok use	0.905	<0.001
13. I use TikTok so much that it has had a negative impact on my work/study	0.892	<0.001
14. I feel depressed when I do not use TikTok, which goes when I use it	0.855	<0.001
15. I feel anxious when I do not use TikTok, which goes when I use it	0.927	<0.001

Table S7. Intraclass correlation coefficients for the total score for each factor and the TikTok Addiction Scale.

TikTok Addiction Scale	Intraclass correlation coefficient	95% confidence interval	*p*-value
Total score	0.994	0.984 to 0.998	<0.001
Salience	0.962	0.921 to 0.982	<0.001
Mood modification	0.963	0.918 to 0.983	<0.001
Tolerance	0.992	0.982 to 0.996	<0.001
Withdrawal symptoms	0.930	0.853 to 0.967	<0.001
Conflict	0.990	0.977 to 0.995	<0.001
Relapse	0.992	0.984 to 0.996	<0.001

© 2024 the Author(s), licensee AIMS Press. This is an open access article distributed under the terms of the Creative Commons Attribution License (https://creativecommons.org/licenses/by/4.0)
